# APIM-peptide targeting PCNA improves the efficacy of docetaxel treatment in the TRAMP mouse model of prostate cancer

**DOI:** 10.18632/oncotarget.24357

**Published:** 2018-01-27

**Authors:** Caroline K. Søgaard, Siver A. Moestue, Morten B. Rye, Jana Kim, Anala Nepal, Nina-Beate Liabakk, Siri Bachke, Tone F. Bathen, Marit Otterlei, Deborah K. Hill

**Affiliations:** ^1^ Department of Cancer Research and Molecular Medicine, Norwegian University of Science and Technology (NTNU), Trondheim, Norway; ^2^ Department of Circulation and Medical Imaging, Norwegian University of Science and Technology (NTNU), Trondheim, Norway; ^3^ Department of Laboratory Medicine, Women’s and Children’s Health, Norwegian University of Science and Technology (NTNU), Trondheim, Norway; ^4^ Department of Pharmacy, Faculty of Health Sciences, Nord University, Namsos, Norway; ^5^ Department of Radiology, St. Olavs Hospital, Trondheim University Hospital, Trondheim, Norway; ^6^ APIM Therapeutics A/S, Trondheim, Norway; ^7^ Clinic of Surgery, St. Olavs Hospital, Trondheim University Hospital, Trondheim, Norway

**Keywords:** magnetic resonance imaging, gene expression, apoptosis, MAPK, PI3K/AKT

## Abstract

Docetaxel is the chemotherapeutic choice for metastatic hormone-refractory prostate cancer, however, it only marginally improves the survival rate. The purpose of the present study was to examine if a peptide targeting the cellular scaffold protein PCNA could improve docetaxel’s efficacy. We found that docetaxel given in combination with a cell penetrating peptide containing the AlkB homolog 2 PCNA interacting motif (APIM-peptide), reduced the prostate volume and limited prostate cancer regrowth *in vivo* in the immunocompetent transgenic adenocarcinoma model of prostate cancer (TRAMP). In accordance with this, we found that the APIM-peptide enhanced the efficacy of docetaxel *in vitro*. Gene expression analysis on prostate cancer cell lines indicated that the combination of docetaxel and APIM-peptide alters expression of genes involved in cellular signaling, apoptosis, and prostate cancer development. These changes were not detected in single agent treated cells. Our results suggest that targeting PCNA and thereby affecting multiple cellular pathways simultaneously has the potential to improve docetaxel therapy of advanced prostate cancer.

## INTRODUCTION

Prostate cancer (PCa) is the second most common form of cancer among men worldwide, and ranks as the fifth leading cause of cancer death [1]. Generally, chemotherapy treatment remains the last line of therapy for hormone-refractory and metastatic PCa, where docetaxel in combination with prednisone is the most common regimen [2]. Docetaxel’s primary mode of action is to inhibit microtubule disassembly by binding to β-tubulin, leading to inhibition of multiple cellular processes including vesicular transport, transcription factor trafficking, cellular signaling, and inhibition of mitotic cell cycle progression. PCa cells *in vivo* do not necessarily proliferate very rapidly, and promotion of apoptosis and inhibition of androgen receptor transcriptional activity are important non-mitotic effects suggested to be the main reasons why taxanes are the only class of cytotoxic agents to prolong survival in PCa [3]. Still, docetaxel treatment is not curative because drug resistance develops; novel treatment options that improve outcome in advanced PCa are therefore in demand. Combining docetaxel with novel drugs that complement its mode of action could potentially delay the development of resistance. Inhibition of kinase pathways such as the PI3K/Akt/mTOR and mitogen-activated protein kinase (MAPK) pathways, frequently found to be upregulated in PCa, are suggested strategies [4, 5].

PCNA (proliferating cell nuclear antigen), an essential scaffold protein best known for its roles in DNA replication and DNA repair, has emerged in the last decade as an interesting drug target (reviewed in [6, 7]). Recently, it has become evident that PCNA also functions as a scaffold outside the nucleus and is important for regulation of vital cellular mechanisms such as apoptosis [8, 9], immune invasion in cancer cells [10, 11], glycolysis [12], and cellular signaling involving the PI3K/Akt/mTOR and MAPK pathways [13]. These newly discovered functions of PCNA are cell cycle independent (for a recent review see [14]).

PCNA may potentially interact with more than 500 cellular proteins, as these contain either of the two identified PCNA-interacting motifs, the PCNA-interacting peptide (PIP)-box [15] and the AlkB homologue 2 PCNA-interacting motif (APIM) [16]. The PIP-box is found in essential proteins involved in replication, while several proteins involved in DNA repair and DNA damage tolerance mechanisms contain APIM [16–20]. Additionally, multiple proteins including kinases and regulators of apoptosis, contain putative APIM or PIP-box motifs, which suggests that targeting PCNA may impair multiple cellular pathways simultaneously [16]. It has been shown that targeting PCNA with an APIM-peptide impaired cellular defense mechanisms and major signaling pathways, with the consequence of hypersensitivity of cancer cells to chemotherapies *in vitro* and *in vivo* [13, 21, 22]. Interestingly, normal cells were much less affected, and the peptide had low overall cytotoxicity *in vivo*.

Here, we raise the question as to whether targeting PCNA could improve the efficacy of docetaxel in the spontaneous mouse TRAMP PCa model. This model was selected because it recapitulates both the histological characteristics and the progressive development of human PCa towards androgen-insensitivity [23], and it is regarded as a clinically relevant *in vivo* model to evaluate novel therapeutic strategies. PCa growth in the TRAMP model was monitored using magnetic resonance imaging (MRI) *in vivo*. Interestingly, APIM-peptide in combination with docetaxel reduced the tumor regrowth rate compared with docetaxel only, and vehicle treated mice. The combination also reduced growth of four PCa cell lines, one established from a TRAMP-mouse, and three from human PCa. Microarray analysis of the three human cell lines treated with the combination of docetaxel and APIM-peptide showed that the response was different in an androgen-insensitive (PC3 and Du145) compared with androgen-sensitive (LNCaP) background. However, expression of multiple genes commonly dysregulated in PCa was changed in the androgen-insensitive cells, and several of these support the anti-cancer activity observed in the TRAMP mice.

## RESULTS

### APIM-peptide targeting PCNA reduces the regrowth rate of docetaxel treated prostate cancer *in vivo*

**Figure 1:**
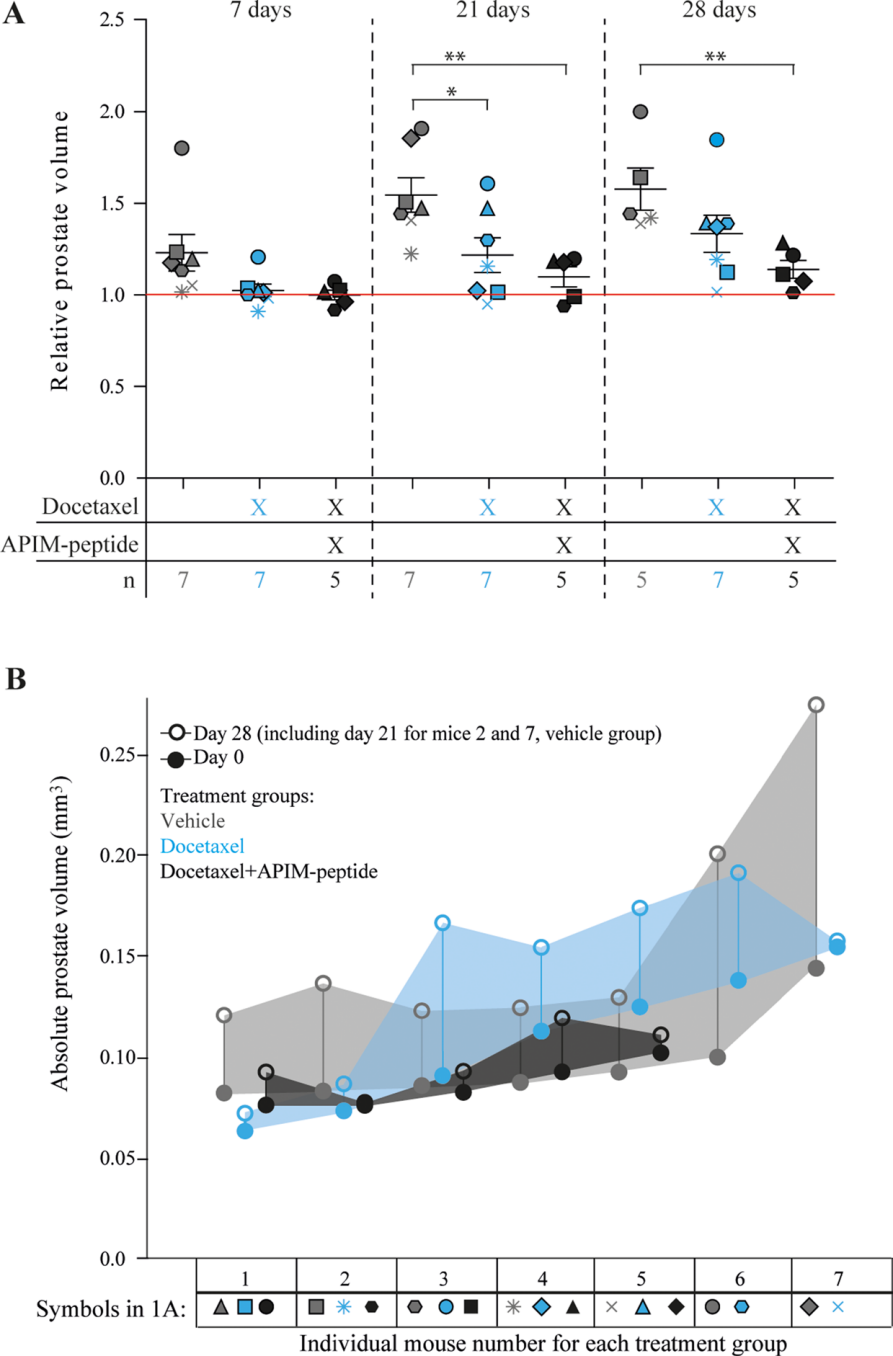
Reduced regrowth rates of prostate cancers in mice when combining APIM-peptide with standard docetaxel treatment (**A**) Prostate volumes at day 7, 21 and 28 after treatment relative to day 0 (day of treatment). Mice were treated on day 0 with vehicle (0.14% (V/V) ethanol in PBS (*n* = 7 on day 7 and 21, *n* = 5 day on day 28, grey symbols), docetaxel (3 mg/kg, 1 dose on day 0, *n* = 7, blue symbols) and docetaxel in combination with APIM-peptide (docetaxel (3 mg/kg) and APIM-peptide (6 mg/kg), 1 dose on day 0, APIM-peptide (6 mg/kg) on days 2 and 3, *n* = 5, black symbols). Each individual mouse is represented by a different symbol and color denotes treatment group. The average ± S.E.M. are displayed with bars in each group. The red line represents the prostate volume before treatment at day 0. Statistical significance/*p*-values were calculated by an unpaired, two-tailed student *t*-test. Prostate volumes of the two sacrificed mice from the vehicle group on day 21 are included in the average weight on day 28 as they represent a minimum. *p* < 0.05, ^*^p < 0.005^**^. (**B**) Absolute prostate volumes of individual mice are shown for day 0, closed circles, and day 28 (day 21 for mice 2 and 7, vehicle group), open circles. Different colours denote the different treatment groups. The shaded area allows visualization of the absolute prostate growth across the group. Symbols corresponding to each individual mouse in 1A are shown below the individual mouse number.

We investigated whether an increased anti-cancer effect could be observed *in vivo* in the TRAMP model of PCa by combining docetaxel with the PCNA targeting APIM-peptide. MRI was used to determine prostate volume immediately before the first treatment (day 0), and again at days 7, 21, and 28 (Figure 1A and B). A significant increase in the relative prostate volume was observed in vehicle treated mice at day 7, but not in the docetaxel or the combination treated groups, indicating an effect of both treatments (Figure 1A). On day 7, docetaxel and combination groups showed similar drug responses based on tumor volumes. By day 21 the combination group showed a trend towards slower tumor regrowth compared with both vehicle and docetaxel groups, and this trend was maintained at day 28. At day 21, a significant difference in prostate volume between vehicle and docetaxel, and between vehicle and combination groups was observed. Two mice in the vehicle group were terminated due to unacceptable tumor burden at day 21. By day 28 there was only a significant difference between combination and vehicle groups, suggesting that the docetaxel group experienced increased cancer regrowth compared to the combination group. Additionally, the combination treatment led to a more uniform response across the individual mice (Figure 1A), i.e. the spread of the data was greater for docetaxel treatment alone. Initial dose-response studies supported reduced relative prostate volume in combination groups compared to docetaxel groups (Supplementary Figure 1). As previous studies have indicated low or no single agent efficacy and low toxicity of the APIM-peptide in various murine cancer models [21, 22] (and unpublished), we did not include an APIM-peptide single agent group in this study. The low single agent activity of the APIM-peptide implies that the increased effect of the combination treatment compared with docetaxel alone is likely synergistic.

There was no significant difference between absolute prostate volumes between any of the groups at day 0, whereas by day 28, combination treated mice were significantly smaller than vehicle treated. Thus, response to therapy was not influenced by variations in starting prostate volume (Figure 1B, Supplementary Figure 1B). Cell proliferation analysis with Ki67 staining on TRAMP prostate tissue at day 28 indicated slightly reduced proliferation in treated prostates compared to vehicle treated prostates, however, there was no significant difference between the groups (Supplementary Figure 2). The main prostate regrowth in the docetaxel group occurred from day 7 to day 21, and then stagnated from day 21 to day 28, likely explaining why no difference between docetaxel and combination groups could be detected at the end point. The combination treated mice did not have higher weight loss than the docetaxel only treated mice (data not shown), suggesting that APIM-peptide increased the efficacy of docetaxel without reducing the well-being of the mice. This is in accordance with observations in other *in vivo* studies with APIM-peptides in combination with DNA damaging chemotherapeutics [13, 21, 22].

### APIM-peptide potentiates the efficacy of docetaxel in prostate cancer cell lines and modifies the gene expression

**Figure 2:**
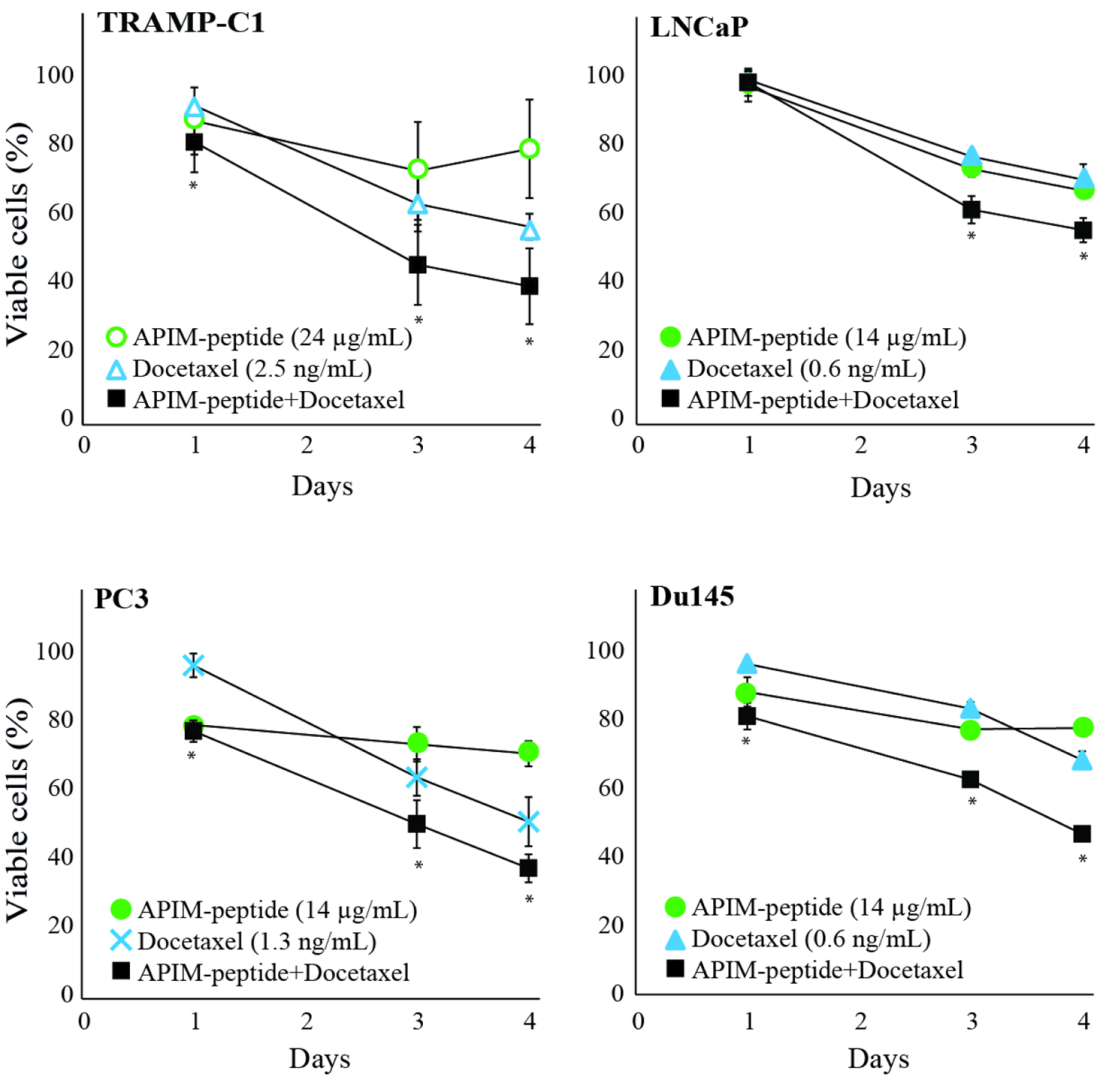
APIM-peptide further reduces the growth of docetaxel-stressed prostate cancer cells Percentage viable TRAMP-C1, PC3, Du145, and LNCaP cells relative to untreated cells (100%). Viability measured by MTT assay after continuous exposure from day 0 until day 4 to APIM-peptide (14 μg/mL and 24 μg/mL, green circles)), docetaxel (0.6 ng/mL, 1.3 ng/mL and 2.5 ng/mL, blue triangles or crosses) or the corresponding combinations of APIM-peptide and docetaxel (black squares). The doses shown were the lowest doses of each agent that had effect as single agents, or displayed an enhanced effect when combined. Mean ± S.E.M. from three independent biological replicas are plotted. Significant differences between the combination and docetaxel groups were calculated by a one sided non-parametric Wilcoxon Sign Rank Test, and are marked with ^*^.

Different cancer cell lines, from a variety of tissue origins, have different sensitivity against the APIM-peptide [21, 22]. In general, cancer cell lines are more sensitive than normal cell lines, and primary cancer cells are more sensitive than primary normal cells. Here, we examined whether APIM-peptide could enhance the growth inhibitory efficacy of docetaxel in two androgen-sensitive (LNCaP and TRAMP-C1) and two androgen-insensitive (Du145 and PC3) PCa cell lines [24]. The murine cell line TRAMP-C1 (derived from a TRAMP tumor) was less sensitive than the human cell lines, both to docetaxel and APIM-peptide. However, an increased efficacy in the combination group compared to docetaxel alone was seen in all four cell lines (Figure 2).

**Figure 3:**
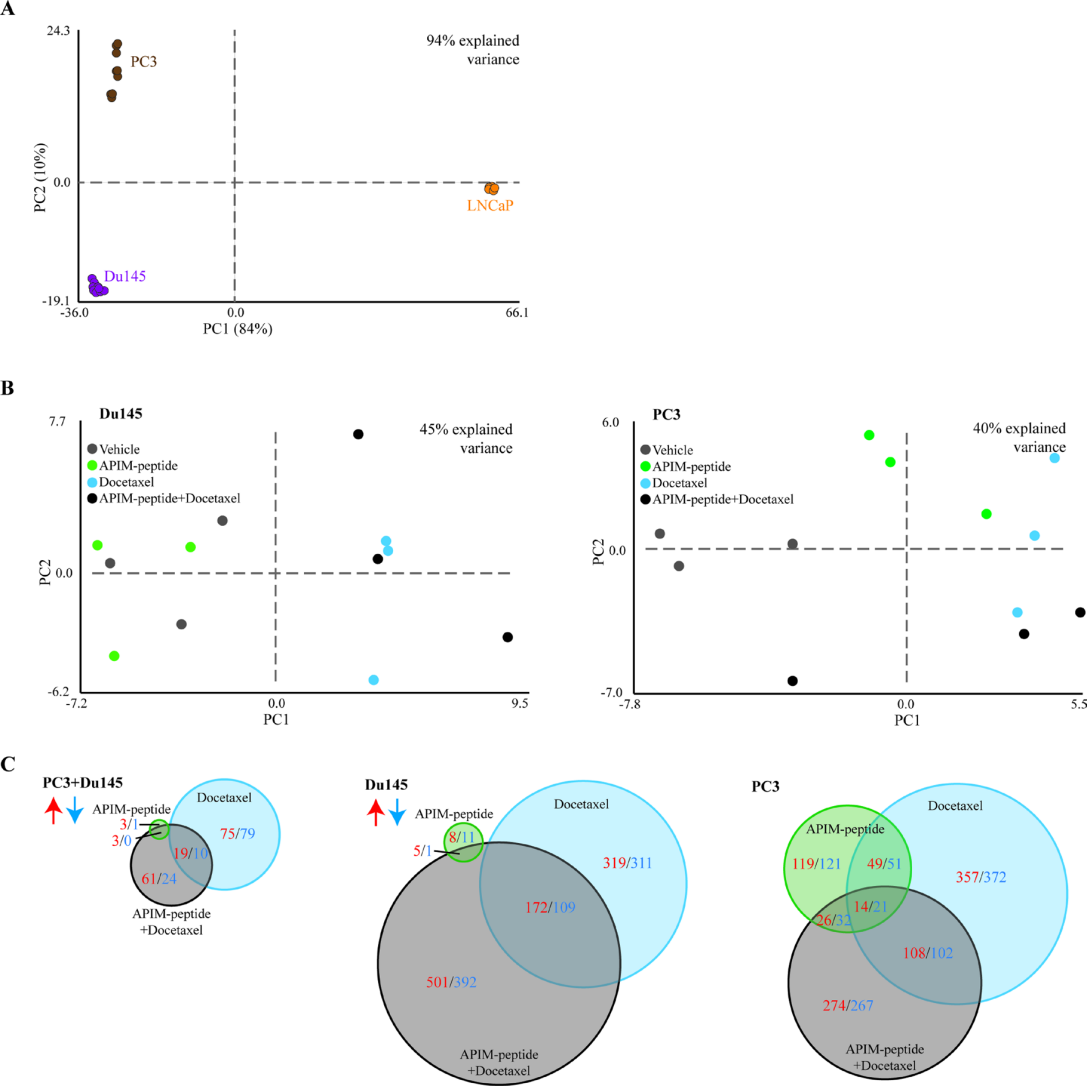
Response to drug treatment is cell-line specific, and combination of APIM-peptide with docetaxel modifies gene expression Microarray analysis on PC3, Du145 and LNCaP human prostate cancer cells treated with APIM-peptide (14 μg/mL APIM-peptide, green), docetaxel (1.3 ng/mL (Du145 and LNCaP) or 2.5 ng/mL (PC3), blue) or the combination of these (black) for 24h (*n* = 3 for each treatment of each cell line). Principal component analysis (PCA) identifies the most pronounced variation modes in the data by combining the original data variables (here genes) into more condensed variables, termed principal components. PCA plots of differentially expressed (DE) genes from all treatments relative to untreated (*n* = 9 for all cell lines: 3 biological replicas, 3 treatment groups). PCA displaying general differences between (**A**) PC3 (brown), Du145 (purple) and LNCaP (orange) cell lines, (**B**) treatment groups in Du145 and PC3 cells after correction of replica variations. (**C**) Venn diagram of number of DE genes significantly upregulated (red) or downregulated (blue) in each treatment group relative to untreated control common in both PC3 and Du145 cells (left panel) or separately in Du145 cells (middle panel) and PC3 cells (right panel) (*n* = 6, 3 biological replicas for each cell line).

To further explore the molecular mechanisms of the APIM-peptide induced increase in docetaxel efficacy, we concentrated on the three human PCa cell lines, and analyzed changes in gene expression under similar treatment regimes. Principal component analysis (PCA) showed that the largest variation in gene expression patterns was attributed to differences between the three human cell lines, rather than differences between the treatments (Figure 3A). The androgen-sensitive LNCaP cell line was the main contributor to the variation, while the androgen-insensitive Du145 and PC3 cell lines had more similar expression patterns. While the data points were clustered for LNCaP cells, a larger spread was observed for Du145, and especially for PC3 cells. However, the largest variations within the cell lines were attributed to the different replicas. By correcting for replica variations, we looked for differences in gene expression between the treatment groups in Du145 and PC3 cells (Figure 3B). In Du145 cells, vehicle and APIM-peptide treatments were separated from docetaxel and combination treatments, while in PC3 cells each treatment was grouped more separately, indicating differences between treatment groups on a gene expression level.

No significant differentially expressed (DE) genes (relative to untreated control) were common across all three combination treated cell lines. By excluding LNCaP, however, several DE genes were shared between PC3 and Du145 cells. While the APIM-peptide as single drug did not alter gene expression, clear effects of the docetaxel and combination treatments could be detected (Figure 3C, left panel). However, looking at the changes in the PC3 and Du145 cell lines separately, it is clear that the PC3 cell line responded more to the APIM-peptide alone than Du145 (Figure 3C). Viability of PC3 was also more affected than Du145 at 24 hours by the APIM-peptide single treatment (Figure 2).

The APIM-peptide in combination with docetaxel affects the expression of multiple genes in both Du145 and PC3 cells that are not influenced by docetaxel as a single agent. Interestingly, several of the affected genes are commonly dysregulated in PCa, a few selected genes are shown in Table 1, and a comprehensive list is shown in Supplementary Table 1. For example, the drug combination, but not single treatments, downregulated expression of RPS6KA2, LY6E, NUSAP1, NUAK2 and PAICS, five genes reported to promote PCa development and progression [25–31]. Additionally, the drug combination upregulated PINK1, IRF1, PPP1R15A, CRABP2, SCRN1, LIMA1, and TGFBI; all genes associated with tumor suppression functions in PCa [32–41]. These changes could be contributing factors to the increased anti-cancer effect observed when combining APIM-peptide with docetaxel in the TRAMP-model. Functional enrichment analysis of the DE genes by the combination treatment found in both cell lines (grey circle in Figure 3C) did not point towards one specific cellular response/pathway, but showed that genes involved in multiple cellular responses, including responses to stress and DNA repair, were affected (Supplementary Table 2).

**Table 1:** APIM-peptide in combination with docetaxel affects gene expression of genes commonly dysregulated in prostate cancer

Downregulated							
Gene	Reference to prostate cancer	PC3			Du145		
		Rank	Log2 (FC)	*P*-val	Rank	Log2 (FC)	*P*-val
Ribosomal Protein S6 Kinase 90 kDa Polypeptide 2 (RPS6KA2)	[25, 49]	219	−0.2	0.016	489	- 0.2	0.020
Nucleolar And Spindle Associated Protein 1 (NUSAP1)	[29]	57	−0.2	0,0018	390	−0.2	0.0150
Lymphocyte Antigen 6 Complex, Locus E (LY6E)	[27]	83	- 0.2	0.004	684	- 0.2	0.028
NUAK Family, SNF1-like Kinase 2 (NUAK2)	[28]	410	- 0.1	0.032	777	- 0.1	0.034
Phosphoribosylaminoimidazole Carboxylase And Phosphoribosylaminoimidazolesuccinocarboxamide Synthase (PAICS)	[31]	94	−0.1	0.0049	949	−0,2	0.0481
**Upregulated**							
PTEN Induced Putative Kinase 1 (PINK1)	[32, 61]	141	0.2	0.009	162	0.2	0.003
Interferon Regulatory Factor (IRF1)	[33, 62]	331	0.2	0.027	301	0.2	0.010
Protein Phosphatase 1, Regulatory Subunit 15A (PPP1R15A)	[36, 63]	74	0.3	0.003	103	0.3	0.001
Cellular Retinoic Acid Binding Protein 2 (CRABP2)	[37]	188	0.2	0.014	343	0.2	0.013
Secernin 1 (SCRN1)	[35]	12	0.2	0.0002	217	0.2	0.006
LIM Domain and Actin Binding 1 (LIMA1)	[41]	98	0.2	0.005	214	0.2	0.006
^*^Transforming Growth Factor Beta Induced 68 kDa (TGFBI)	[39]	10	0.5	0.00005	101	0.4	0.001

Selection of significant (*p* < 0.05) differentially expressed (DE) genes from microarray analysis found in both Du145 and PC3 cells (*n =* 6) treated with the combination of APIM-peptide (14 μg/mL) and docetaxel (1.3 ng/mL (Du145) or 2.5 ng/mL (PC3)). The selected genes listed were only found in the combination treatment, and are commonly dysregulated in human prostate cancer. The downregulated genes listed are reported to be tumor promoting and the upregulated genes listed are reported to be tumor suppressing in given publications. The complete list of all DE genes is presented in Supplementary Table 1. FC = Fold change. ^*^More enhanced (as described by p-value and rank) in combination than docetaxel treatment.

### Combining APIM-peptide with docetaxel alters cellular signaling and increases apoptosis in prostate cancer cell lines

**Figure 4:**
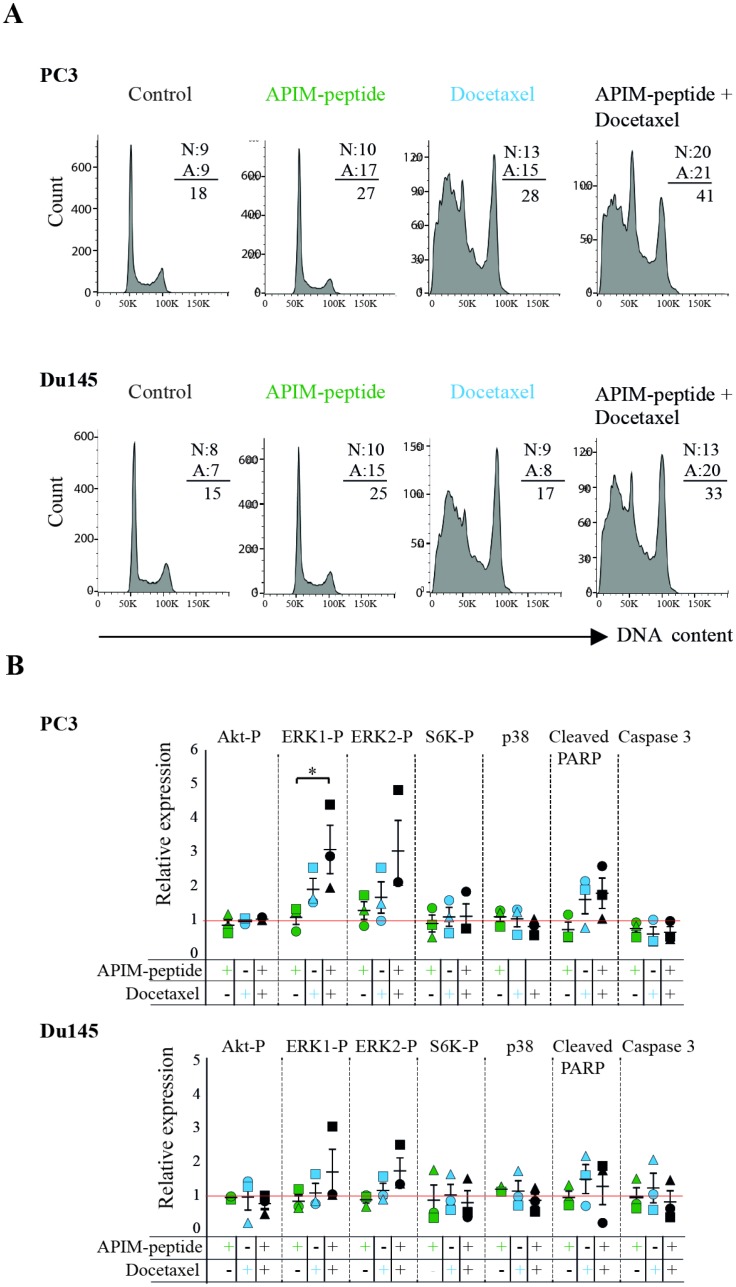
APIM-peptide increases the effects of docetaxel on apoptosis and cellular signaling (**A**) Cell cycle analysis and fraction of apoptotic (A) vs necrotic (N) cells (values from contour plots are given). (**B**) Relative expression of phosphorylated Akt, ERK1, ERK2, S6K, and p38, cleaved-PARP, and caspase 3. For all experiments, PC3 and Du145 cells were treated with APIM-peptide (14 μg/mL, green), docetaxel (2.5 ng/mL: PC3; 1.3 ng/mL: Du145; 5 ng/mL, blue) and the combination of these (black) for 24 hours prior to the analysis. The protein levels (B) were adjusted for loading differences (β-tubulin) and normalized against untreated cells, and additionally for total protein levels for the phosphorylated proteins. Data are from three biological replicas (different symbols represents extracts acquired on different days). Statistically significant differences (^*^p < 0.05) were calculated by a Kruskal-Wallis H test, and a post-hoc Dunn’s test was used to determine which groups this applied to.

Several proteins in the PI3K/Akt and MAPK pathways contain the APIM sequence and the APIM-peptide affects the activation of these pathways during stress [13]. These signaling pathways are important regulators of cell proliferation and apoptosis, therefore we next examined whether APIM-peptide alone, or in combination with docetaxel, altered apoptosis, cell cycle, and central kinases. We detected an increased level of apoptotic (Annexin V-positive, A) cells in both PC3 and Du145 cultures treated with APIM-peptide as a single agent, while the fractions of necrotic (PI-positive, N) cells were unchanged (results from contour plots are given as numbers in Figure 4A). Combination of docetaxel and APIM-peptide increased the fraction of both apoptotic and necrotic cells compared to the single treatments. The cumulative percentage of dead cells (A + N) were 33% and 41% in Du145 and PC3, respectively, thus approximately an increase of 10% relative to single agent treatments and 20% relative to untreated control. APIM-peptide treatments did not affect the cell cycle distribution in any of the cell lines tested, neither alone nor in combination with docetaxel. Docetaxel alone, on the other hand, strongly affected cell cycle distribution and/ or the cellular DNA content (G2/M peak increased) in accordance with its mode of action (Figure 4A).

In general, the changes in kinases and apoptotic factors detected by western analysis were small and for the most part not significant, however some trends could be observed. APIM-peptide as a single agent did not affect the level or activation of the signaling proteins tested (Figure 4B), which is in accordance with small changes in gene expression after treatment with APIM-peptide alone (Figure 3C and ArrayExpress, E-MTAB-4858). When combined with docetaxel, however, an increase in ERK1 and 2 phosphorylation was observed relative to untreated control. Also, a tendency towards reduced p38 levels in the combination treated cells compared to docetaxel alone, and reduced phosphorylation of S6K, acting downstream of Akt, was observed in two out of three biological replicas in both cell lines (Figure 4B). Flow cytometry analysis indicated that APIM-peptide treatment increased the levels of apoptosis, so we also examined the extracts for the apoptotic markers cleaved-PARP and caspase 3. Reduced full-length caspase 3 and increased levels of cleaved-PARP were observed in most replicas of docetaxel and combination treated cells in both cell lines. In summary, these results identified some changes in proteins important for apoptosis and cellular signaling in the combination treated cells, not seen or seen at a lower level in the single agent treated cells.

## DISCUSSION

There are limited treatment options available for patients with PCa progressing after docetaxel treatment. Prednisone in combination with carbazitaxel may be the most promising option and is suggested to slightly increase the survival of these patients; it is however, associated with increased toxicities [42–44]. To address new treatment options we monitored PCa regrowth in the TRAMP model following treatment with a combination of docetaxel and a peptide targeting PCNA. TRAMP mice as young as 10 weeks of age display androgen-insensitive characteristics [23], and the mice used in this study (25–28 weeks old) are therefore representative of androgen-insensitive PCa. Analysis of two human androgen-insensitive cell lines revealed initial changes in gene expression, apoptosis, and cellular signaling that supported the reduced regrowth detected in combination treated mice.

Development of drug resistance to both chemotherapeutics and targeted therapies are a major problem; combining targeted drugs with chemotherapy or targeting multiple signaling pathways simultaneously, have been suggested to combat this problem [45]. Given PCNA’s role as a scaffold protein affecting multiple signaling pathways involved in regulation of metabolism, apoptosis, DNA repair and cell cycle, and its overexpression in several cancers, PCNA has emerged as a potential target in cancer therapy [6, 7]. PCNA interacts with numerous proteins via its two PCNA interacting motifs, the PIP-box and APIM, which have overlapping binding sites on PCNA [17, 21]. Multilayered regulatory mechanisms determine which proteins interact with PCNA at a given time. This includes affinity driven competition, posttranslational modifications (PTMs) on PCNA or the PCNA binding proteins, half-life/ stability of proteins and the context of the PCNA complexes, e.g. repair vs. replication, nuclear vs. cytosolic and normal growth vs. stress situations. Different PIP-box variants have up to 700 fold different affinity for unmodified PCNA in absence of DNA (reviewed in [7, 46, 47]). Affinity differences between APIM consensus variants are also likely, but APIM variants have low affinity towards PCNA involved in replication, as stable cells overexpressing APIM-peptide are viable and proliferate normally, while PIP-box expressing cells die. APIM-peptide overexpressing cells are however, hypersensitive to cellular stress [16, 48]. Normal cells are largely unaffected by the peptide both *in vitro* and *in vivo* [13, 21]. In agreement with low affinity for PCNA involved in replication we see no/ low effects on the cell cycle for APIM-treated cells in Figure 4A. The APIM-peptide therefore seems to mainly impair PCNA scaffold functions important for cellular stress mechanisms.

Enhancing the efficacy of docetaxel if combined with the APIM-peptide could reduce the dose needed, reducing the discomfort for the patients and potentially extending the duration of chemotherapy. It is challenging to pinpoint the most prominent pathway that is impaired by the combination treatment in TRAMP-mice as it is likely that a multitude of pathways are affected. In addition, because these tumors occur spontaneously, one explanation does not necessarily fit for all of the mice, or even for all the tumor cells within one tumor. Nevertheless, gene expression data from the two androgen-insensitive cell lines gave some indications as to why the combination therapy had an increased anti-cancer effect in the TRAMP model. RPS6KA2, one of the genes found to be downregulated only in combination treated cells, encodes a serine/threonine kinase acting downstream of MAPK and Akt. Previous studies have described RPS6KA2 as a diagnostic PCa marker [25] and it contains one out of eleven single nucleotide polymorphisms (SNPs) associated with PCa risk [49]. Furthermore, RPS6KA2 displayed increased expression in PCa tissue compared to normal tissue, and inhibition lead to decreased proliferation of PCa cells [25]. Synthetic lethality between RPS6KA2 and erlotinib (EGFR inhibitor) is shown in PCa, supporting extensive crosstalk between MAPK and Akt pathways in PCa, and illustrates the clinical potential for combinatory treatments affecting these pathways [50]; these pathways are also affected by the APIM-peptide. Interestingly, a recent publication on PCa progression has pinpointed the importance of NUSAP1, one of the genes downregulated by the combination treatment [29]. NUSAP1 is an important microtubule-associated protein [51]; its role in PCa is suggested to be via induction of FAM101B that modulates cell shape and is involved in the TGF-β pathway promoting tumor invasion. NUSAP1 knockout resulted in reduced migration in PCa cell lines, reduced tumor volume in PCa xenografts, and a NUSAP1 knockdown gene expression signature correlated with better outcome in patient PCa samples [29]. Moreover, NUSAP1 is the only gene that appears in two out of the three commercially available gene expression signatures currently used for assessing aggressive PCa [52, 53] and (Rye et al, unpublished). Another frequently overexpressed gene in PCa, LY6E, was revealed as downregulated by microarray analysis in combination treated cells [27]. Increased expression of LY6E, an interferon inducible gene, is shown to be linked to poor survival in multiple cancers. LY6E promotes signaling via the TGF-β pathway leading to increased drug resistance, PDL1 and CTLA4 expression, and immune escape in breast cancer [54]. The APIM-peptide is shown to affect several of the downstream mediators of TGF-β such as the ERKs, p38, TNF-α and PI3K/Akt, as well as interferon which act upstream of LY6E [13, 55]. Microarray analysis also revealed a downregulation of two other frequently overexpressed genes in PCa, NUAK2 and PAICS [28, 31]. NUAK2 (SNARK) is a NF-kappaB-regulated anti-apoptotic gene [56], while PAICS is a *de novo* purine biosynthetic enzyme. A recent study identified PAICS as essential for PCa cell growth and progression; its knockdown caused reduced PCa growth both *in vitro* and *in vivo*, thus PAICS was indicated as an important target in PCa treatment [31]. The downregulation of these genes could be related to the APIM-peptide’s ability to modulate members of the upstream pathways, such as MAPKs and PI3Ks.

Several interesting genes were also found to be upregulated by the combination treatment. The exact molecular mechanism behind the increased expression is not known, but cellular signaling networks are highly dynamic and the ripple effects extensive. In any case, a reduced expression of these genes is reported to be associated with increased cancer cell growth and/or invasiveness (see references in Table 1). Therefore, an increased expression of these genes may support the reduced re-growth observed in the TRAMP model.

In summary, the effects observed in growth assays, western, flow cytometry, and gene expression analysis support the increased efficacy seen in the TRAMP mouse model when docetaxel is given in combination with the APIM-peptide. Multiple proteins involved in cellular signaling, including kinases, phosphatases, and ubiquitin ligases, contain the APIM or the PIP-box PCNA binding sequence, thus indicating a central role for PCNA as a cytosolic scaffold [16]. Small changes in multiple pathways are expected when inhibiting the abilities of PCNA-interacting proteins to bind to their scaffold, which is likely to be the reason for the modified docetaxel response observed in combination with the APIM-peptide. Even with modest changes, if the combination of the APIM-peptide and docetaxel could result in the same clinical outcome using a lower docetaxel dose, then there could be noticeable improvements to patient quality of life from reduced docetaxel-induced side effects. In support of this, in a pilot study in the TRAMP model using a low dose of docetaxel (0.5 mg/kg versus 3 mg/kg in the main study) a significant increase in initial response in the combination treated group compared to docetaxel single treated group was observed (Supplementary Figure 1). In all our experiments, we treated the mice for only one week; in future experiments, however, it would be of interest to administer repeated weekly treatments with low dose docetaxel in combination with the APIM-peptide to look at long-term effects.

Our results suggest that APIM-peptide in combination with docetaxel downregulated several genes considered to be therapeutic targets, and upregulated several genes with a protective role against PCa. Signaling and gene expression regulation, however, are dynamic processes; the western, flow, and gene expression analysis were only performed 24 hours after treatment, thus limiting our study to a snapshot. Nevertheless, the results clearly demonstrate altered cellular processes, which could explain the regrowth characteristics that we observed in the TRAMP model. For a more complete understanding of the mechanisms underlying the increased anti-cancer efficacy observed by combining APIM-peptide with docetaxel, additional studies and verifications are needed.

In conclusion, our preclinical data supports that APIM-peptide targeting PCNA has the potential to improve the non-mitotic effects of docetaxel. Despite the intrinsic heterogeneity within the TRAMP model, as within PCa patients, APIM-peptide in combination with docetaxel had a marked effect *in vivo*. Thus, this combination has a potential for clinical translation.

## MATERIALS AND METHODS

### Animals and ethics

Animal care and experiments were carried out in accordance with Norwegian and EU guidelines for care and use of laboratory animals, and were approved by the Norwegian National Animal Research Authority and the Norwegian Food Safety Authority (FOTS application 6681). The colony of TRAMP mice used as model organism in all animal experiments, were genetically modified from C57BL/6 mice (Jackson Labs, USA) and established in-house (NTNU, Norway). Genotyping was performed by PCR. The animals were kept in a standardized environment, and monitored for general health status and body weight for the duration of the experiments.

### *In vivo* study design

The mice were recruited to the study at 25–28 weeks old, and mice exhibiting poorly-differentiated tumors on the first magnetic resonance imaging (MRI) exam were excluded from the study [57]. The animals were randomly distributed into three treatment groups: (a) vehicle (0.14% (V/V) ethanol in PBS, day 0, 1, and 2, *n* = 7), (b) docetaxel (3 mg/kg, one dose, day 0, *n* = 7) and (c) docetaxel (3 mg/kg, one dose, day 0) + APIM-peptide (6 mg/kg, three doses, day 0, 1, and 2, *n* = 5). In accordance with the reduction principle of the 3Rs (reduce, refine, replace), we reduced the number of mice in the combination treatment group; we anticipated a higher tumor burden in the vehicle and docetaxel groups, thus increasing the likelihood of earlier termination and the requirement of more animals. An APIM-peptide single drug treatment group was not included in the study because previous studies have demonstrated that APIM-peptide as a single agent did not reduce tumor growth *in vivo* [21, 22]. Treatments were performed by intraperitoneal injections. Prostate growth was assessed by MRI at day 7, 21 and 28 after treatment. The animals were terminated at day 28 after treatment, or when MRI revealed an unacceptable tumor burden.

### MRI

MRI was performed on a 7T scanner (Biospec 70/20 Avance III, Bruker Biospin MRI, Ettlingen, Germany) with a volume resonator (86 mm diameter) for RF transmission and a phased array mouse heart surface coil for reception. Mice were anesthetised (~2% isoflurane in medical air with 36% O2) for the duration of the MRI scan and positioned on the scanner bed in a prone position. Breathing motion in the pelvic region was reduced by firmly securing the mouse to the scanner bed with adhesive tape across its lower back. The respiration rate was monitored (SA Instruments, USA) and the body temperature was kept (37°C) by circulating warm water through the bed. For full details on the MRI imaging sequences and parameters, see [57], in brief, the following imaging sequences were performed: Low-resolution T2 weighted (LR-T2W) images were acquired in axial and coronal planes using a RARE spin echo sequence to check correct positioning of the mouse. High-resolution T2W (HR-T2W) images were acquired in the axial plane using a RARE spin echo sequence. Diffusion weighted (DW-MRI) images were acquired using a Stejskal-Tanner prepared multi-shot EPI sequence with b-values = 0, 100, 200, 400, 800 along three orthogonal gradient directions over the same region of the mouse as HR-T2W images to allow for image registration. ADC maps were calculated in MatLab (MathWorks, Natick, MA) by voxelwise fitting of the signal (S) averaged over all gradient directions using a monoexponential model for all b-values according to S(b)=S_0_exp(−b ADC), where S_0_ is the signal intensity for b0.

### MRI assessment of prostate volume

Whole prostate volumes (including ventral, lateral and dorsal lobes) from each mouse were calculated by manually-drawn ROIs based on HR-T2W images using OsiriX (Pixmeo SARL, Switzerland), employing b800 DW-MR images as a reference to discriminate prostate from seminal vesicle (SV), according to the method described in [57]. Data are reported as mean ± S.E.M. and statistical significance between groups was calculated using an unpaired, two-tailed Student’s *t*-test (*p* < 0.05).

### Treatment agents

APIM-peptide (ATX-101), a 25 amino acid cell penetrating peptide containing the APIM sequence (APIM Therapeutics, Norway) [21] and docetaxel (Actavis, Iceland).

### Cell lines

One PCa cell line isolated from TRAMP mice (TRAMP-C1, ATCC-CRL-2730) and three human PCa cell lines, PC3 (ATCC CRL-1435, androgen-insensitive, high metastatic potential) Du145 (ATCC HTB-81, androgen-insensitive, moderate metastatic potential), and LNCaP (androgen-sensitive, low metastatic potential) were used for the *in vitro* studies. LNCaP cells were kindly provided by Senior Engineer Berit Størdal, Department of Molecular Medicine, Norwegian University of Science and Technology, Norway. All cell lines were grown in DMEM or RPMI-1640 medium (Sigma-Aldrich, UK) supplemented with fetal bovine serum (10%, Sigma-Aldrich, Norway), amphotericin B (2.5 μg/mL, Sigma-Aldrich, MO, USA), L-glutamine (2 mM, Sigma-Aldrich, MO, USA) and penicillin (100 units/mL)-streptomycin (0.1 mg/mL) (Gibco, NY, USA). Cells were cultivated in a humidified atmosphere (95% air, 5% CO_2_, 37°C).

### Cell viability assay

Cell growth over time was measured using the 3-(4.5-Dimethylthiazol-2-yl)-2.5 diphenyl-tetrazolium bromide (MTT, Sigma-Aldrich, MO, USA) assay similarly as in [16]. Cells were seeded in 96-well plates (3,000 cells/well) and continuously exposed to APIM-peptide (14 μg/mL APIM-peptide for Du145, PC3 and LNCaP, or 24 μg/mL for TRAMP-C1 and docetaxel (0.6 ng/mL for Du145 and LNCaP, 1.3 ng/mL for PC3, or 2.5 ng/mL for TRAMP-C1) until harvest at day one, three, and four. The ratio of viable cells in individual replicas of docetaxel and APIM-peptide + docetaxel treated cells versus untreated cells were log transformed with base 2. The resulting values reflecting the change of docetaxel + APIM-peptide relative to docetaxel only were subjected to one sided non-parametric Wilcoxon Signed Rank Test as implemented in MATLAB R2015a (MathWorks Inc.), in order to check if the combination treatment further reduced the viability compared to docetaxel alone.

### Cell treatments for flow cytometry analysis, western blot and microarray

Cells were seeded in plates (3 million cells/15 cm plate) the day before treatment; APIM-peptide (14 μg/mL for Du145, PC3 and LNCaP, or 24 μg/mL for TRAMP-C1) (same dose as in the MTT assay) and docetaxel (1.3 ng/mL for Du145 and LNCaP, 2.5 ng/mL for PC3, or 5.0 ng/mL for TRAMP-C1) (2× doses compared to MTT assay, see below) were given as single agents or in combination (three treatment groups and one untreated control group per cell line). Twice as many cells/ area were used compared to the MTT assay and experiments showed that twice as high docetaxel dose was needed to obtain the same effect with respect to viability, while the same APIM-peptide dose gave similar effects. The cells were exposed continuously for 24 hours before harvest. Three biological replicas started on different days (*n* = 3) were prepared for all groups of each cell line.

### Cell extracts

Cell extracts were prepared as previously described [13]. Briefly, cells were collected, resuspended and incubated in lysis buffer (1.5 h, 4°C), followed by sonication (2 min, 2.5 output control, 20% duty cycle) and centrifugation (10 min, 4°C, 13 600 rpm). The supernatants were collected as total cell extract.

### Flow cytometry analysis

The fraction of apoptotic cells and the cell cycle distribution were analyzed. 1 million live cells were labeled with the Dead Cell Apoptosis Kit (Molecular Probes) according to the manufacturer’s protocol. Briefly, the cells were resuspended in 100 μl annexin-binding buffer (10 mM HEPES, 140 mM NaCl, 2.5 mM CaCl2, pH 7.4), supplied with 5 μl of Annexin V Alexa Fluor^®^ 488 and 1 μl of Propidium Iodide (PI; 0,1 mg/ml), incubated for 15 minutes at room temperature and analyzed on a BD FACS Canto flowcytometer (BD Biosciences). Cells stained with Annexin V Alexa Fluor^®^ 488 and PI were excited with a blue laser (488 nm), and the Annexin V Alexa Fluor^®^ 488 fluorescence and the PI fluorescence were detected in the FITC (530/30; 502LP) and the PE (585/42; 556LP) channel, respectively. The fraction of apoptotic (A) and necrotic (N) cells was determined by using the FlowJo, LLC software (USA), and the numbers are given in the histograms showing DNA contents (described below). Parallel samples of cells were fixed in ice-cold 100 % methanol and stored at 4°C until cell cycle analysis. The cells were washed with cold phosphate-buffered saline (PBS) and incubated with 200 μl of DNase-free RNAse A (Sigma) in PBS (100 μg/ml) for 30 min at 37°C before DNA staining with 200 μl of PI (Sigma) (50 μg/ml) at 37°C for 30 min. Cell cycle analyses were performed by using a BD FACS Canto flowcytometer (BD Biosciences). PI stained cells were excited with the blue laser (488nm), and the PI fluorescence was detected in the PE channel. Cell cycle fractions were determined by using the FlowJo, LLC software.

### Western blot analysis

Cell extracts (50 μg) were added to LDS loading buffer (1×) and DTT (0.1 M) and incubated (10 min, 70°C) to reverse cross-links. Proteins were separated by electrophoresis (10% Bis-Tris gels, NuPAGE, Invitrogen, CA, USA) and subsequently transferred to polyvinylidene fluoride membranes (Immobilon, Millipore, Ireland). The membranes were blocked in blocking buffer (5% dry milk in PBS) before incubation with primary antibodies against Akt (Cell Signaling Technology, #4691, MA, USA), Phospo-Akt (P-Akt, Ser473, Cell Signaling Technology, #4060, MA, USA), ERK1/2 (ERK, Santa Cruz Biotechnology, sc-93-G, TX, USA), Phospho-ERK1/2 (P-ERK, Thr202/Tyr204, #4370, Cell Signaling Technology, MA, USA), p70 S6 Kinase (S6K, Cell Signaling Technology, #2708, MA, USA), Phospho-S6K (P-S6K, Thr389, Cell Signaling Technology, #9206, MA, USA), p38 (Abcam, ab31828, UK), cleaved PARP (Abcam, ab4830, UK), Caspase 3 (Cell Signaling Technology, #9662, MA, USA), β-tubulin (Abcam, ab6046, UK) and β-actin (Abcam, ab8226, UK). The fluorescently labelled secondary antibodies goat α-rabbit 680RD, goat α-mouse 800CW and donkey α-goat 800 CW (all Li-Cor Biosciences, UK) were used for protein detection. All antibodies were diluted in blocking buffer (5% dry milk in PBS with 0.1% Tween 20). The proteins were visualized in Odyssey infrared imaging system (LI-COR Biosciences, UK) and quantified in Odyssey Image Studio (V2.0). Protein levels were normalized against β-tubulin or β-actin and compared to untreated control. Phosphorylated proteins were additionally compared to the total protein levels. Data are reported as mean ± S.E.M. Significant differences (*p* <0 .05) between groups were calculated using a Kruskal-Wallis H test in Matlab (MathWorks, Natick, MA, USA) and a post-hoc Dunn’s test [58] was used to determine which groups were significantly different.

### Microarray

Cells were harvested (2.5 × 10^6^ cells) and total RNA extracted using the RNeasy mini kit (QIAGEN, Germany). Genome-wide gene expression profiling was performed by using Illumina HumanHT-12 v4 Expression BeadChip (Illumina Inc. CA, USA), providing a coverage of more than 24,000 annotated genes (47,231 probes corresponding to 1 to 3 probes per gene) including well characterized genes and splice variants. Using the Illumina TotalPrep RNA Amplification Kit from Ambion (Thermo Fisher Scientific Inc. MA, USA), extracted total RNAs (500 ng) were converted to cDNAs and subsequent biotin labeled single-stranded cRNAs. Prior to cRNA synthesis the integrity of the total RNA was analyzed using the Agilent 2100 bioanalyzer with the Agilent RNA 6000 Nano Kit. The concentration of cRNA was measured with NanoDrop 8000 UV-Vis Spectrophotometer instrument (Thermo Scientific, DE, USA) and normalized (150 ng/μl). Biotin labeled cRNAs (1.5 μg) were hybridized overnight to the HumanHT-12 Expression BeadChips. Subsequent steps included washing, streptavidin-Cy3 staining and scanning of the arrays were performed on an Illumina HiScan instrument. Probe and intensity data were exported from Illumina’s proprietary software. The microarray experiments are minimum information about a microarray experiment (MIAME) compliant and have been deposited in the ArrayExpress database (http://www.ebi.ac.uk/arrayexpress/) under accession number E-MTAB-4858.

### Gene expression analysis

Prior to differential expression analysis, probes with low detection (*p* < 0.1) were filtered, data log2- transformed, and quantile normalized. Log2-transformed expression values for all samples (*n* = 36 (four groups: Docetaxel, APIM-peptide, combination, untreated), three biological replicas conducted on separate days for three cell lines) were subjected to principal component analysis (PCA) [59]. PCA identifies the most pronounced variation modes in the data by combining the original data variables (here genes) into more condensed variables termed principal components. To emphasize differences between the different groups, baseline expression level differences due to variation between cell-lines and biological replicates were minimized by subtracting the mean expression value from each subset of replicates in each cell line (nine subsets in total), which was done for each gene. Differentially expressed (DE) genes were calculated by the limma package in R, and identified *p*-values were adjusted for multiple hypothesis testing using Benjamini and Hochberg false discovery rate (FDR) [60]. Gene changes specific to the combination treatment were defined as those DE (*p* < 0.05) in the combination treatment of both Du145 and PC3, but not DE when treated with docetaxel in neither Du145 nor PC3. In addition, genes with increased rank (by *p*-value) and fold-change (FC) in the combination treatment of both Du145 and PC3 compared to docetaxel were identified by manual inspection of the ranked gene lists, and defined as enhanced by the combination treatment.

## SUPPLEMENTARY MATERIALS FIGURES AND TABLES




