# Supplementary material for: APIM-peptide targeting PCNA improves the efficacy of docetaxel treatment in the TRAMP mouse model of prostate cancer
Source: Oncotarget. 2018 Jan 27;9(14):11752–66. doi: 10.18632/oncotarget.24357 (PMC5837745; doi:10.18632/oncotarget.24357)
Supplement: Supplementary file 1 [file oncotarget-09-11752-s001.pdf]

APIM-peptide targeting PCNA improves the efficacy of docetaxel treatment in the TRAMP mouse model of prostate cancer

SUPPLEMENTARY MATERIALS

Histology and immunostaining of TRAMP prostate tissue

Upon sacrifice of the TRAMP mice, the genitourinary (GU) tract (prostate, SV, emptied bladder) was excised, weighed, and fixed in formalin (10%) for at least 48 hours. Samples were embedded such that the sectioning plane was aligned with the MRI images, as described in (Hill et al., 2015); DW-MRI images were used to identify cancerous areas, which appeared bright in comparison to the surrounding prostate tissue, and histology sections were acquired for these regions. Formalin fixed paraffin embedded samples were sectioned (4 µm slice thickness) and stained with haematoxylin (ChemiTeknik AS, Norway) erythrosine B (Sigma-Aldrich, Norway) and saffron (ChemiTeknik AS, Norway) using an automatic slide stainer (Sakura Tissue-Tek© Prisma™). Additional sections were acquired for Ki67 immunostaining; de-waxed sections (4 µm) were pretreated in Target Retrieval Solution, High pH (Dako K8004) in PT Link (Dako) (20 minutes, 97°C) to facilitate antigen retrieval. The further staining was performed on Dako Autostainer: following washes in wash buffer (Dako S3006), endogenous peroxidase activity was quenched by incubation in Peroxydase block (Dako K4007). Sections were then washed in wash buffer and blocked with Protein Block Serum-Free (Dako X0909) (20 minutes). Excessive reagent was removed, and the slides were incubated with primary antibody Ki-67 (abcam ab16667, clone SP6 1:50) (40 minutes). The slides were then washed in wash buffer and incubated (30 minutes) in Labelled polymer HRP anti-Rabbit (K4007) and with DAB to develop the stain. Slides were lightly counterstained with hematoxylin using an automatic slide stainer (Sakura Tissue-Tek© Prisma™), dehydrated through ascending grades of ethanol, cleared in Tissue Clear and coverslipped. For the tissue studied, appropriate negative controls were performed by omitting the primary antibody.

Cell proliferation *in vivo*

Cell proliferation in prostate tissue was assessed by Ki67 staining after harvesting the GU tracts (28 days after treatment, except for two mice treated with vehicle that were harvested day 21) from mice treated with vehicle (n = 7), docetaxel (n = 7), or combination (n = 5). 40× magnification images were obtained from an Olympus BX41 microscope with an Olympus DP26 camera; images were exported using labSens software (Olympus, 2010), and imported into OsiriX (Pixmeo SARL, Switzerland), where nuclei were manually counted. The samples were anonymized before counting. The number of positive and negative stained nuclei were recorded, with a minimum of 500 nuclei per region; counting was performed separately for regions of normal proliferation, and high proliferation (hotspot) on each sample. Owing to variation in the tissue morphology between prostate lobes in mice, ventral prostate was chosen to define proliferation in normal regions. Hotspot regions occurred in the lateral or dorsal prostate. The proliferation index was calculated as the percentage of positively stained nuclei for each region.

Supplementary Table 1: APIM-peptide combined with docetaxel treatment results in DE genes not found in docetaxel single agent treatment. See_Supplementary_Table 1

Supplementary Table 2: Functional enrichment analysis on differentially expressed genes by the APIM-peptide and docetaxel combination treatment

Term	BH	ES
**Downregulated DE genes**		
Annotation cluster 1		3.65
Nuclear lumen	1.2E-3	
Non-membrane-bounded organelle	2.1E-3	
Intracellular non-membrane-bounded organelle	2.1E-3	
Annotation cluster 2		3.20
Replication fork	1.1E-4	
Cellular response to stress	1.1E-3	
Nucleotide-excision repair	6.1E-3	
Annotation cluster 3		1.80
ATP-binding	2.1E-2	
Nucleotide-binding	2.2E-2	
Nucleoside-binding	3.6E-2	
**Upregulated DE genes**		
Annotation cluster 1		3.73
Cellular macromolecular complex subunit organization	1.7E-5	
Cellular macromolecular complex assembly	3.2E-5	
Histone core	4.1E-5	
Annotation cluster 2		2.67
Cellular macromolecular complex subunit organization	1.7E-5	
Tubulin/FtsZ, GTPase domain	3.2E-5	
Tubulin	3.2E-5	

(Related to Figure 3C (left pnael, grey group). The gene name IDs of the DE genes detected in both combination treated PC3 and Du145 cells were submitted to database for annotation, visualization and integrated discovery (DAVID) for functional enrichment analysis. The table lists the enrichment score (ES) from the significant annotation clusters from downregulated and upregulated DE genes, and the top three (as ranked by Benjamini-Hochberg, BH) significant gene ontology terms in each annotation cluster.

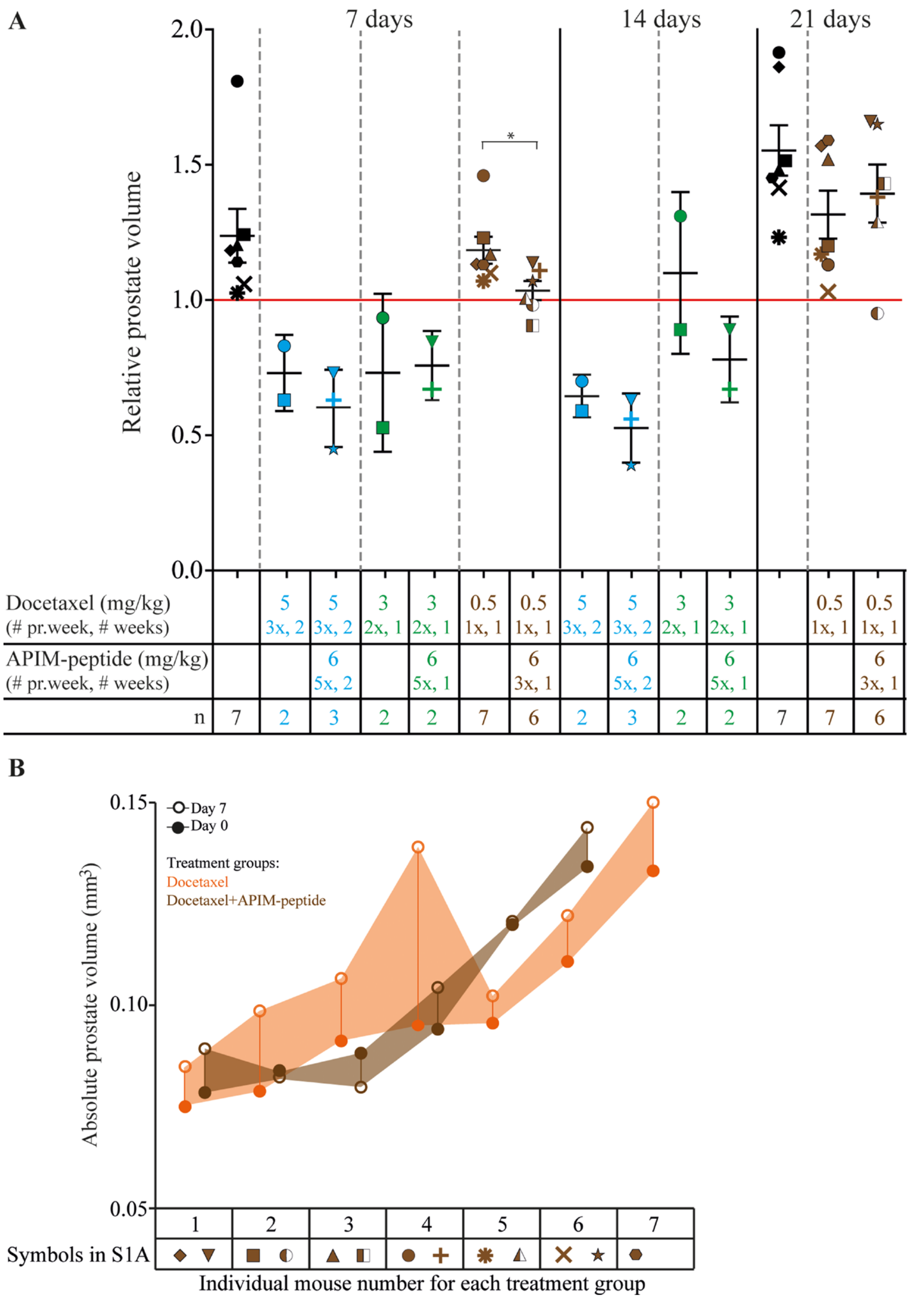

Supplementary Figure 1: Initial dose-response studies show reduced prostate volumes in TRAMP mice when combining APIM-peptide with docetaxel treatment. (**A**) Prostate volume at day 7 and 14 or 21 after treatment relative to day 0 (day of treatment). Mice were treated on day 0 with with vehicle (0.14% (V/V) ethanol in PBS), a variation of docetaxel doses (0.5-5 mg/kg, 1-3x/week, 1-2 weeks) or docetaxel in combination with APIM-peptide (6 mg/kg APIM-peptide, 3-5x/week, 1-2 weeks). The details of the treatment regimens as well as the number of mice in each group (n) are shown in the scheme below the graph. Different colors mark each treatment regimen (docetaxel and combination). Each individual mouse is presented by a different symbol. The average ± S.E.M. ($n > 3$) or SD ($n \leq 3$) are displayed with bars in each group. The red line represents the prostate volume before treatment at day 0. Statistical significance/p-values were calculated by an unpaired, two-tailed student t-test, $p < 0.05$ *. Vehicle group (black) are from the main study displayed in Figure 1. The initial and highest docetaxel dose tested (5 mg/kg docetaxel, blue) indicated reduced prostate volume for both treatment groups, but an increased efficacy in the combination group compared to docetaxel group. By reducing the docetaxel dose (3 mg/kg, green), both treatment groups had an initial response to therapy, while at day 14 only the docetaxel group experienced regrowth. The lowest docetaxel dose tested (0.5 mg/kg docetaxel, brown) showed an initial response in the combination group but not the docetaxel group, the difference between the treatment groups were significant (day 7), but at day 21 no difference was detected. The pilot experiments support our findings that the APIM-peptide increases the efficacy of docetaxel. (**B**) Absolute prostate volumes of individual mice treated with low-dose docetaxel (0.5 mg/kg) as single agent (orange) or in combination with APIM-peptide (6 mg/kg, as in main study) (brown) before treatment at day 0 (closed circles) and after treatment at day 7 (open circles). The shaded area allows visualization of the absolute prostate growth across the group. Symbols corresponding to each individual mouse in S1A are shown below the individual mice number.

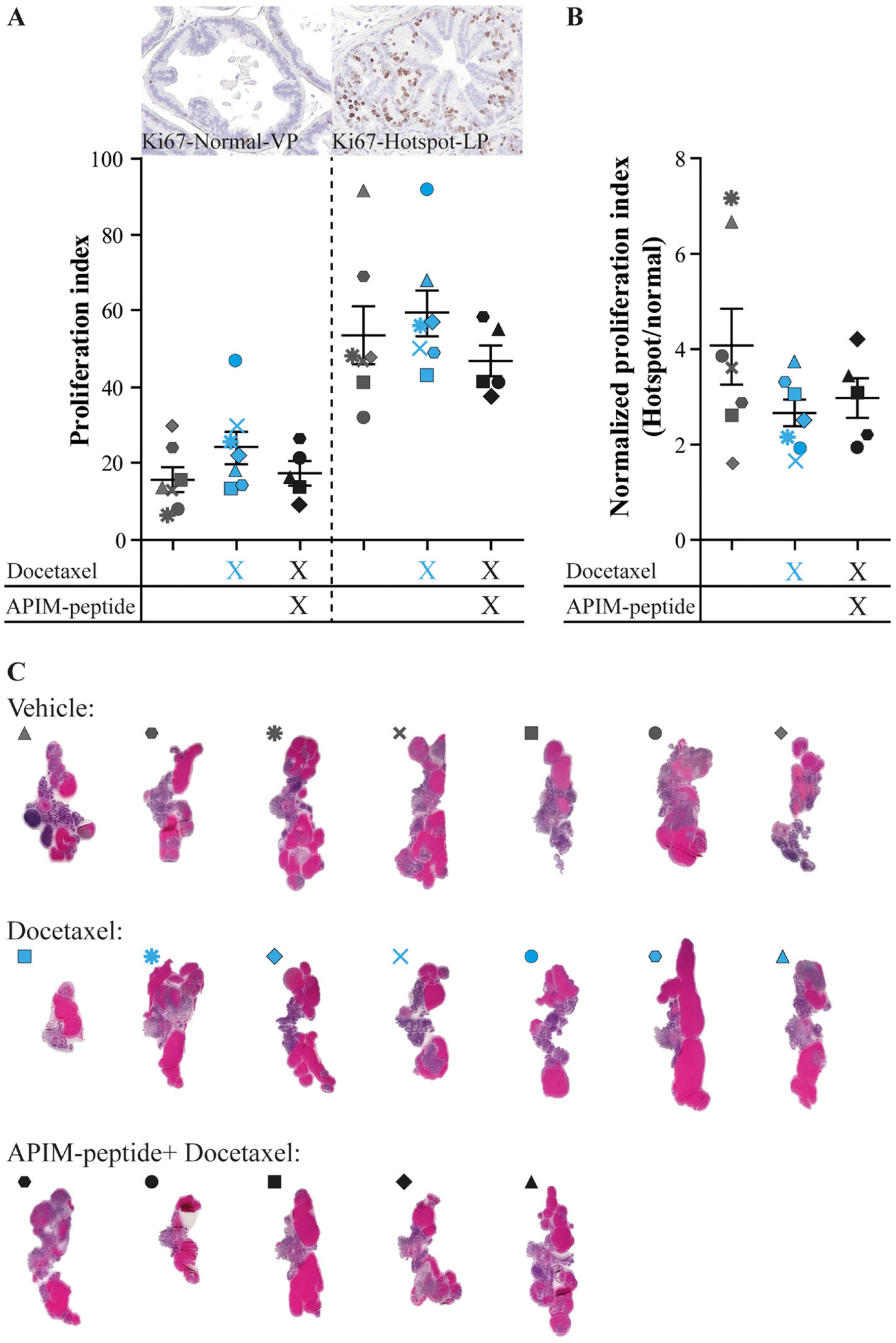

Supplementary Figure 2: Ki67 results at day 28 demonstrate cell proliferation *in vivo*. Prostate tissue harvested 28 days after treatment with vehicle (0.14% (V/V) ethanol in PBS, *n* = 7, grey symbols, vehicle prostates presented with diamond and triangle symbols were harvested at day 21), docetaxel (3 mg/kg, 1 dose on day 0, *n* = 7, blue symbols) and docetaxel in combination with APIM-peptide (docetaxel (3 mg/kg) and APIM-peptide (6 mg/kg), 1 dose of both on day 0, 2 additional doses of APIM-peptide on day 2 and 3, *n* = 5, black symbols). Symbols represent individual mice, and are matched with those in Figure 1 for reference. VP = ventral prostate. LP = Lateral prostate. For experimental procedures, see Supplementary materials and methods (below). (**A**) Proliferation index (percentage positive Ki67 cells) for normal and hotspot regions of TRAMP prostate tissue. Representative Ki67 histology images from a control animal at 40× magnification are included. (**B**) Normalized Ki67 proliferation index (hotspot/normal for each mouse). (**C**) Photographs of HES stained GU tracts from all mice. HES sections were acquired immediately adjacent to the Ki67 sections (data shown in (Supplementary Figure 2B)). The large pink regions are seminal vesicles; prostates are generally centrally located. The images are to deomonstrate that mice included in this study were all exhibiting well-differentiated adenocarcinoma, and none exhibited neuroendocrine carcinoma.
