# Supplementary material for: APIM-peptide targeting PCNA improves the efficacy of docetaxel treatment in the TRAMP mouse model of prostate cancer
Source: Oncotarget. 2018 Jan 27;9(14):11752–66. doi: 10.18632/oncotarget.24357 (PMC5837745; doi:10.18632/oncotarget.24357)
Supplement: Supplementary file 2 [file oncotarget-09-11752-s002.docx]

**Supplementary Table 1: APIM-peptide combined with docetaxel treatment results in DE genes not found in docetaxel single agent treatment** (related to Table 1).

Significant (p<0.05) DE genes (relative to untreated control) in both PC3 and Du145 (n=6) identified in the APIM-peptide + docetaxel combination treated cells, but not in docetaxel treated cells.

| **Downregulated** | | | | | | | | |
| --- | --- | --- | --- | --- | --- | --- | --- | --- |
| **Gene name** | **GeneCard description** | **PC3** | | | | **Du145** | | |
|  |  | **Rank** | **FC** | **P-value** | | **Rank** | **FC** | **P-value** |
| Myosin Light Chain Kinase (MYLK) | Implicated in smooth muscle contraction and cytoskeletal rearrangements. | 256 | -0.2 | 0.0201 | | 96 | -0.3 | 0.0011 |
| Nucleolar And Spindle Associated Protein 1 (NUSAP1) | Plays a role in spindle microtubule organization. | 57 | -0.2 | 0.0018 | | 390 | -0.2 | 0.0150 |
| Synemin (SYNM) | Cytoskeletal protein important in muscle. | 200 | -0.2 | 0.0141 | | 648 | -0.1 | 0.0273 |
| DNA Polymerase, Delta 3 (POLD3) | The 66 kDa subunit of DNA polymerase delta crucial in DNA replication and DNA repair. | 374 | -0.2 | 0.0304 | | 309 | -0.2 | 0.0108 |
| Purine-Rich Element Binding protein B (PURB) | Single stranded DNA binding protein implicated in DNA replication and transcription. | 259 | -0.2 | 0.0203 | | 434 | -0.2 | 0.0173 |
| Synovial Sarcoma Translocation, Chromosome 18 (SS18) | Appear to have a function as a transctiptional coactivator. | 497 | -0.2 | 0.0429 | | 467 | -0.2 | 0.0193 |
| Ribosomal Protein S6 Kinase 90 kDa Polypeptide 2 (RPS6KA2) | Serine/threonine kinase implicated in signaling controlling cell survival and growth. | 219 | -0.2 | 0.0159 | | 489 | -0.2 | 0.0201 |
| Phosphoribosylaminoimidazole Carboxylase And Phosphoribosylaminoimidazolesuccinocarboxamide Synthase (PAICS) | Catalyzes steps 6 and 7 of purine biosynthesis. | 94 | -0.1 | 0.0049 | | 949 | -0.2 | 0.0481 |
| NUAK Family, SNF1-like Kinase 2 (NUAK2) | Stress-activated kinase involved in tolerance to glucose starvation. | 410 | -0.1 | 0.0324 | | 777 | -0.1 | 0.0341 |
| Annexin A7-Like protein 2 (ANXA8L2) | May function as an anticoagulant. | 287 | -0.2 | 0.0229 | | 348 | -0.2 | 0.0127 |
| DQ893812 | No description. | 11 | -0.3 | 0.0002 | | 43 | -0.2 | 0.0003 |
| C15orf48 | No description. | 59 | -0.3 | 0.0018 | | 48 | -0.3 | 0.0004 |
| Lymphocyte Antigen 6 Complex, Locus E (LY6E) | No description. | 83 | -0.2 | 0.0037 | | 684 | -0.2 | 0.0281 |
| 5-Nucleaotidase Domain Containing 1 (NT5DC1) | No description. | 551 | -0.2 | 0.0473 | | 525 | -0.2 | 0.0217 |
| *Replication Factor C Subunit 5 (RFC5) | The smallest subunit of the replication factor C complex, which is required for DNA replication and for loading of proliferating cell nuclear antigen onto DNA during S phase. | 136 | -0.2 | 0.0087 | | 298 | -0.2 | 0.0102 |
|  | | | | | | | | |
| **Upregulated** | | | | | | | | |
| Lysine Specific Demethylase 2B (KDM2B) | Histone demethylase important for transctiptional repression for inhibition of cell growth and proliferation. | 243 | 0.2 | | 0.01918 | 843 | 0.1 | 0.03920 |
| Tetraspanin 9 (TSPAN9) | Transmembrane protein probably mediating signal transduction for cell growth and motility. | 519 | 0.1 | | 0.04425 | 939 | 0.1 | 0.04758 |
| PTEN Induced Putatice Kinase 1 (PINK1) | Serine/threonine kinase in mitochondria important for protection of mitochondrial dysfunction during stress. | 141 | 0.2 | | 0.00889 | 162 | 0.2 | 0.00293 |
| Interferion Regulatory Factor (IRF1) | Transcriptional activator of genes induced by interferon α. β and γ with a potential role in tumor-suppression and apoptosis. | 331 | 0.2 | | 0.02698 | 301 | 0.2 | 0.01031 |
| Protein Phosphatase 1. Regulatory Subunit 15A (PPP1R15A) | Iimportant in cellular stress. Downregulates TGF-β signaling. and may promote apoptosis. | 74 | 0.3 | | 0.00310 | 103 | 0.3 | 0.00122 |
| Jun Proto-Oncogene (JUN) | Regulates gene expression. | 30 | 0.4 | | 0.00094 | 56 | 0.4 | 0.0004 |
| Ubiquitin-Conjugating Enzyme E2H (UBE2H) | Involved in ubiquitination of proteins for degradation. | 247 | 0.2 | | 0.01959 | 55 | 0.3 | 0.00043 |
| P21 Protein (CDC42/Rac)-Activated Kinase 2 (PAK2) | Serine/threonine kinase involved in several signaling pathways. Activated during caspase-dependent apoptosis. and may thus have a role in apoptosis. | 139 | 0.3 | | 0.00865 | 427 | 0.2 | 0.0164 |
| Cell Division Cycle 27 (CDC27) | Component of the anaphase promoting complex/cyclosome (APC/C). that controls progression through mitosis by mediating ubiquitination and degradation of target proteins. | 218 | 0.3 | | 0.01576 | 510 | 0.3 | 0.0213 |
| Cyclin Dependent Kinase Inhibitor 2C (CDKN2C) | Interacts with CDK4/6 to inhibits cell growth and proliferation with a dependence retinoblastoma protein RB. | 498 | 0.2 | | 0.04295 | 662 | 0.2 | 0.0275 |
| Chloride Intracellular Channel 3 (CLIC3) | Can form chloride ion channels. May participate in cellular growth control. | 481 | 0.2 | | 0.04130 | 60 | 0.4 | 0.0006 |
| Makorin Ring Finger Protein 1 (MKRN1) | E3 ubiquitin ligase promoting degradation of target proteins. Keeps cells alive by suppressing p53/TP53 under normal conditions. but stimulates apoptosis by repressing CDKN1A under stress conditions. | 225 | 0.2 | | 0.01652 | 764 | 0.2 | 0.0326 |
| Trafficking Protein Particle complex 6B (TRAPPC6B) | May be involved in vesicle transport. | 170 | 0.2 | | 0.01059 | 854 | 0.1 | 0.04021 |
| Tubulin Alpha 1C (TUBA1C) | One of the major subunits of microtubules. a cytoskeletal component. important in cellular structur. transportation and cell division. | 36 | 0.2 | | 0.00107 | 362 | 0.2 | 0.01361 |
| Tubulin Beta 2A (TUBB2A) | Microtubule component important in mitosis and intracellular transport. | 13 | 0.3 | | 0.00023 | 901 | 0.2 | 0.0444 |
| Tubulin Beta 2B (TUBB2B) | Microtubule compontent. | 3 | 0.4 | | 0.000009 | 371 | 0.2 | 0.0140 |
| Elastin Microfibril Interfacer 2 (EMILIN2) | May be involved in cell adhesion. | 252 | 0.2 | | 0.01969 | 634 | 0.2 | 0.0269 |
| LIM Domain and Actin Binding 1 (LIMA1) | Cytoskeleton-associated protein inhibiting actin filament depolymerization. | 98 | 0.2 | | 0.00504 | 214 | 0.2 | 0.0063 |
| FXYD Domain Containing Ion Transport Regulator 5 (FXYD5) | Involved in down-regulation of E-cadherin which results in reduced cell adhesion. Promotes metastasis. | 378 | 0.1 | | 0.03081 | 956 | 0.1 | 0.0484 |
| SERTA Domain Containing 1 (SERTAD1) | Act as a coactivator or corepressor regulating E2F-targeted gene expression. | 159 | 0.2 | | 0.00954 | 447 | 0.2 | 0.01772 |
| SERTA Domain Containing 2 (SERTAD2) | Act as a coactivator or corepressor regulating E2F-targeted gene expression. | 69 | 0.2 | | 0.00260 | 772 | 0.1 | 0.03399 |
| TMF1-Regulated Nuclear Protein 1 (TRNP1) | DNA-binding factor regulating gene expression. May be important in cell cycle progression. | 54 | 0.3 | | 0.00173 | 842 | 0.2 | 0.03920 |
| Ankryn-Repeat Domain 1 (ANKRD1) | May function as a transcription factor. | 40 | 0.4 | | 0.00120 | 296 | 0.3 | 0.01008 |
| Histone Cluster 2B H2ab (HIST2H2AB) | Core component of the nucleosome. controlling DNA accessability important for transcription. DNA replication and DNA repair. | 91 | 0.2 | | 0.0048 | 471 | 0.2 | 0.0195 |
| Carbonyl Reductase 3 (CBR3) | Catalyzes the conversion of carbonyl compounds to alcohols. | 438 | 0.2 | | 0.03587 | 378 | 0.2 | 0.01440 |
| Aldehyde Dehydrogenase 1 Family Member A3 (ALDH1A3) | Enzyme with retinal as a substrate. | 297 | 0.2 | | 0.02437 | 405 | 0.2 | 0.01523 |
| TCDD-Inducible Poly(ADP-Ribose) Polymerase (TIPARP) | Involved in the transfer of ADP-ribose onto glutamic acid residues of a protein to alter its function. | 31 | 0.3 | | 0.00094 | 75 | 0.3 | 0.00071 |
| Cytochrome P450 Family 1 Subfamily B Polypeptide 1 (CYP1B1) | Cytochrome P450 enzyme involved in drug metabolism and oxidation of steroids. fatty acids and xenobiotics. | 5 | 0.4 | | 0.00001 | 39 | 0.3 | 0.00027 |
| Cytochrome P450 Family 24 Subfamily A Polypeptide 1 (CYP24A1) | Cytochrome P450 enzyme involved in drug metabolism and vitamin D3 activation. | 174 | 0.3 | | 0.01103 | 77 | 0.4 | 0.00075 |
| Cellular Retinoic Acid Binding Protein 2 (CRABP2) | Involved in retinoid signaling and transportation of retinoic acid to the nucleus. | 188 | 0.2 | | 0.01355 | 343 | 0.2 | 0.0125 |
| Ubiquinol-Cytochrome C Reductase Complex Assembly Factor 1 (UQCC) | Required for the assembly of the ubiquinol-cytochrome c reductase complex . | 220 | 0.2 | | 0.01607 | 911 | 0.1 | 0.0451 |
| CD68 Molecule (CD68) | Promote phagocytosis and activation of macrophages. | 113 | 0.2 | | 0.00719 | 245 | 0.2 | 0.0074 |
| Kruppel-Like Factor 2 (KLF2) | Regulates T-cell trafficking. | 49 | 0.3 | | 0.00157 | 165 | 0.3 | 0.0032 |
| TSC22 Domain Family. Member 3 (TSC22D3) | Plays a role in anti-inflammtory and immunesuppressive effects of glucocorticoids and interleucin 10 (IL-10). | 540 | 0.2 | | 0.04599 | 805 | 0.2 | 0.03747 |
| SMAD Family Member 6 (SMAD6) | Signal transducer participating in transforming growth factor (TGF)-β and bone morphogenetic protein (BMP) anti-inflammatory activity. | 228 | 0.2 | | 0.01761 | 915 | 0.2 | 0.04571 |
| Calcium Binding Tyrosine-Phosphorylation Regulated (CABYR) | May have a function in fertility. | 290 | 0.2 | | 0.02306 | 258 | 0.2 | 0.00820 |
| Neuronal Pentraxin I (NPTX1) | Part of neuronal response system. | 61 | 0.3 | | 0.00205 | 179 | 0.3 | 0.00403 |
| Secernin 1 (SCRN1) | May be involved in regulation of exocytosis. | 12 | 0.2 | | 0.00022 | 217 | 0.2 | 0.0064 |
| Protein Tyrosine Phosphatase Receptor Type F Polypeptide Interactin Protein Alpha 1 (PPFIA1) | May regulated diassembly of focal adhesions. | 268 | 0.2 | | 0.02136 | 384 | 0.2 | 0.0149 |
| FLJ41455 | No description. | 515 | 0.1 | | 0.04425 | 734 | 0.1 | 0.03112 |
| KIAA1644 | No description. | 476 | 0.2 | | 0.04115 | 240 | 0.2 | 0.00722 |
| Inositol 1.4.5-Trisphosphate Receptor Interacting Protein Like 2 (ITPRIPL2) | No description. | 7 | 0.4 | | 0.00002 | 937 | 0.2 | 0.0472 |
|  |  |  |  | |  |  |  |  |
| *Transforming Growth Factor Beta Induced 68 kDa (TGFBI) | Induced by TGF-β and inhibits cell adhesion. | 10 | 0.5 | | 0.000050 | 101 | 0.4 | 0.0012 |
| *TUBB3 | Component of the microtubules involved in axon guidance and maintenance. | 1 | 0.6 | | 8E-06 | 31 | 0.5 | 0.0001 |
